# Barriers to and facilitators of nurse-parent interaction intended to promote healthy weight gain and prevent childhood obesity at Swedish child health centers

**DOI:** 10.1186/1472-6955-12-27

**Published:** 2013-12-05

**Authors:** Susann Regber, Staffan Mårild, Jan Johansson Hanse

**Affiliations:** 1 Nordic School of Public Health NHV, Box 12 133, Gothenburg SE- 402 42, Sweden; 2 Department of Public Health and Community Medicine, Public Health Epidemiology Unit, Sahlgrenska Academy, University of Gothenburg, Gothenburg, Sweden; 3 Department of Paediatrics, Institute of Clinical Sciences, Sahlgrenska Academy, University of Gothenburg, Gothenburg, Sweden; 4 Department of Psychology, University of Gothenburg, Gothenburg, Sweden

**Keywords:** Child, Preschool, Obesity, Health promotion, Prevention, Child health centers

## Abstract

**Background:**

Overweight and obesity in preschool children have increased worldwide in the past two to three decades. Child Health Centers provide a key setting for monitoring growth in preschool children and preventing childhood obesity.

**Methods:**

We conducted semi-structured interviews with 15 nurses working at Child Health Centers in southwest Sweden in 2011 and 2012. All interviews were tape recorded and transcribed verbatim and imported to QSR N’Vivo 9 software. Data were analyzed deductively according to predefined themes using content analysis.

**Results:**

Findings resulted in 332 codes, 16 subthemes and six main themes. The subthemes identified and described barriers and facilitators for the prevention of childhood obesity at Child Health Centers. Main themes included assessment of child’s weight status, the initiative, a sensitive topic, parental responses, actions and lifestyle patterns. Although a body mass index (BMI) chart facilitated greater recognition of a child’s deviant weight status than the traditional weight-for-height chart, nurses used it inconsistently. Obesity was a sensitive topic. For the most part, nurses initiated discussions of a child’s overweight or obesity.

**Conclusion:**

CHCs in Sweden provide a favorable opportunity to prevent childhood obesity because of a systematic organization, which by default conducts growth measurements at all health visits. The BMI chart yields greater recognition of overweight and obesity in children and facilitates prevention of obesity. In addition, visualization and explanation of the BMI chart helps nurses as they communicate with parents about a child’s weight status. On the other hand, inconsistent use and lack of quality assurance regarding the recommended BMI chart was a barrier to prevention, possibly delaying identification of overweight or obesity. Other barriers included emotional difficulties in raising the issue of obesity because it was perceived as a sensitive topic. Some parents deliberately wanted overweight children, which was another specific barrier. Concerned parents who took the initiative or responded positively to the information about obesity facilitated prevention activities.

## Background

Overweight and obesity in preschool children have increased worldwide and currently affects 40 million children younger than five years of age [1]. Moreover, obesity in preschool children correlates strongly with increased risk for obesity in adolescence [2]. This and many other well-documented associated health consequences of obesity [3] call for preventive interventions in early childhood. In Sweden, representative national data from a survey performed in 2008 showed an overweight prevalence of 17% of children aged seven to nine years, including 3% with obesity [4]. No representative national data are available in preschool children. However, in two separate studies, one from the north and one from the south of Sweden, from 2007/2008 and 2003–2008, respectively, showed similar prevalence data of overweight and obesity in four-year-old children. Overweight was present in about 15 to 17% and obesity in 3% of the children [5,6].

Although childhood obesity is common in both rural and urban areas [1], reports suggest that it occurs more frequently in children from lower socioeconomic groups [1,7]. The World Health Organization (WHO) recommends population-based prevention strategies on multiple levels to counteract the epidemic of childhood obesity [1]. Primary health care is a key setting for monitoring health in preschool children and preventing childhood obesity.

In Sweden, Child Health Centers (CHC) date back to 1938, when the Swedish Parliament decided to offer regular and free CHC visits to all children from birth to six years of age [8]. Today, such visits are widely accepted (up to 99.9% between birth and the age of one year) [9]. CHCs are staffed and run autonomously by specialized full-time nurses who are responsible for general health care in cooperation with part-time physicians, who are responsible for the medical aspects of health care. The national program for CHCs includes growth monitoring, vaccinations, disease screening, counseling on feeding practices, accident prevention and promotion of an active childhood and healthy family lifestyle. Children and their parents see CHC nurses 15 or more times compared to three to five visits to a physician. The interaction between nurses and parents on issues related to children’s growth and healthy lifestyle crucially affect impact and outcome.

This study aimed to examine nurses’ perception of the nurse-parent interaction at CHCs and assess barriers to and facilitators of interaction intended to promote healthy weight gain and prevent obesity. Specifically, we aimed to (i) examine how nurses determine or identify a child’s weight status (i.e. normal weight, under- or overweight, and obesity), (ii) determine who (nurse or parent) initiates discussion about a child’s overweight/obesity, (iii) examine whether a child’s overweight/obesity was too sensitive to discuss with the parents; (iv) investigate nurses’ views on parental responses to their child’s overweight/obesity; and (v) describe health promoting actions and prevention of childhood obesity at CHCs.

## Methods

Participating CHCs were managed by the healthcare authority in the county of Västra Götaland. The authority follows two guidelines for the prevention of childhood obesity: (i) a regional CHC program [10] that stipulates repeated longitudinal growth monitoring, focusing especially on children’s BMI at age 4 and 5½ years; and (ii) a regional web-based decision support system that includes an outline of a healthcare and community-wide obesity prevention and treatment program across the life span [11]. CHC nurses record children’s growth in weight and height charts at all visits. It is also recommended to be registered in Body Mass Index (BMI) charts. Because the county of Västra Götaland does not universally implement electronic health records, some CHCs still use paper health records. A special BMI chart for preschool children includes reference data and age and sex-specific cut-off points, established by the International Obesity Task Force, that allow classification of children into weight categories (i.e., underweight, normal weight, overweight and obesity) [12,13].

### Participant selection and data collection

We recruited nurses working at CHCs in Västra Götaland for interviews in 2011 and 2012 using (i) personal contacts made by SR during another study in the region and (ii) written and verbal information distributed to the four child health developers in Västra Götaland. The child health developers represent four geographical areas of the county and are responsible for further training of the nurses and quality assurance of the methods used at the CHCs. At workplace meetings, the child health developers informed nurses about the interview study and interested nurses were later contacted by SR. The management of each CHC consented to the interviews but did not participate in recruitment. Inclusion criteria for participation were: (i) pediatric or district nurses (ii) actively working in the county of Västra Götaland and (iii) participating voluntarily. We contacted a total of 19 nurses, and the final study population comprised 15 nurses. The nurses who declined participation explained they were either overloaded with work, they doubted being able to contribute in this topic or gave the reason that the CHC area had no children with overweight or obesity. All interviewees received information that followed the WHO’s ethical recommendations for qualitative studies [14]. We aimed to include nurses working among a population with diverse demographic characteristics in different parts of the region, and therefore we also avoided recruiting nurses who worked at two separate CHC units in the same area. By selecting CHCs located in rural areas, small and medium-sized municipalities, and one large city with suburban areas, we intended to achieve a variation in our sample to obtain a plurality of nursing experiences originating from a variety of demographic backgrounds (Table 1). All informants were female and had at least two years of professional work experience as CHC nurses. Nine were specialized as district nurses and six were pediatric nurses. Three CHCs were private primary health care and nine were public primary health care.

**Table 1:** Demographic data of the participants, characteristics of organization, and location characteristics

**Participants**		**n**
Female/Male		15/0
Pediatric nurse/District nurse		6/9
Number of years employed at CHC		
2-5		4
6-9		0
10-25		5
26-29		0
30-35		6
**CHC organization**		
Public primary health care/Private		12/3
**Rural and urban characteristics**	**Number of inhabitants**	
Rural area	< 1 500	2
Coastal island	15 000	1
Small and medium-sized communities in the inner parts of the region	9-24 000	3
Communities of proximity to Gothenburg	35-60 000	5
Inner city area and suburbs of Gothenburg	50-58 000	4

CHC, Child Health Center.

Our semi-structured topic guide covered themes related to study questions that were based on earlier research in this field [15-19]. The questions in the topic guide were expressed as follows: How did you experience the handling of a child with overweight or obesity (in the respective order) concerning the following themes; (i) How did you determine or identify the weight status of the child?, (ii) Who took the initiative to discuss overweight/obesity, you or the parent?, (iii) Have you ever found the topic too sensitive to discuss with parents?, (iv) What were the parental responses?, (v) What were the actions that you can take? Because we changed the topic guide only marginally after conducting two pilot interviews in 2010, we included those interviews in the study. The subsequent 13 interviews were held in 2011 and 2012 at the respective nurse’s CHC reception rooms. All interviews, conducted by SR, were held in Swedish, tape recorded and lasted for 27–35 minutes. The Swedish citations were later translated into English, where the aim was to retain the essence as far as possible. The conversational mode of the interview resulted in a few spontaneous questions that were not given to all interviewees. In addition, SR asked four demographic questions regarding professional specialization, number of years of experience as a CHC nurse, description of the catchment area and number of habitants.

### Data analysis

SR transcribed all interviews verbatim and imported the transcription to computer-assisted qualitative data analysis software (CAQDAS) N’Vivo 9, which provided support for storage, coding, memo writing, sorting and analysis [20]. Analysis began as a pre-reflection phase during the interviews and continued during the transcription phase, when back-and-forth listening provided further opportunity for analysis. The transcribed interviews were read several times. The data were coded deductively [21,22] according to the five themes in the topic guide. One theme that emerged (i.e. lifestyle patterns) and its subthemes were analyzed and coded inductively. A combination of a deductive and an inductive approach is common in practice [22,23]. Meaning units with the most empirically visible and obvious information (i.e. manifest information) were chosen. Further, SR condensed codes with similar contents and then categorized them into subthemes and themes (Table 2). JJH and SM read some of the transcripts, and all authors discussed and analyzed the sorted themes, subthemes, condensed meaning units and their quotations. The background of the authors who contributed to the analysis is as follows: SR is a registered pediatric nurse experienced in working with children with obesity. SM is a pediatrician with long professional experience in CHCs; he also serves as a physician in a regional obesity treatment team. JJH is a professor of psychology and public health and has extensive knowledge of methodology.

**Table 2:** An example of the content analysis procedure, from meaning unit to theme

**IP**	**Meaning unit**	**Code**	**Subtheme**	**Theme**
2	“Because I think the BMI chart is *a very good instrument* to show the parents, that this is what it looks like.”	BMI chart: a great tool to use and display	BMI chart	Assessment of the child’s weight status
2	“If you tell the parents that you do this with all children, no one needs to feel singled out, because I think it is important to not feel singled out.”	Used for all: no one is singled out		
14	“Yeah, and then I am not the one who thought this up. It becomes clearer to the parents [to be shown on the BMI curve].”	Not a subjective opinion by the nurse		
7	“I looked at both height and weight charts and the BMI chart, and I also showed both to the parents.”	Uses both diagrams		
7	“It doesn’t take many minutes to show it to the parents (i.e., the BMI chart), and then you have done it.”	Fast and easy		
14	“Well, you know, everyone can’t actually read. We have that problem. But a chart like this, they can understand that if I explain that there is the maximum and this is average and there is the minimum, and so on.”	A very good tool, also for illiterate parents		
15	‘I hardly do not use the BMI chart.’	Is hardly not used at all		

BMI, body mass index; IP, interview person.

### Ethics

Our study followed Swedish law regarding the ethical review of research involving humans (2003:460) [24]. Ethical approval was not required because we did not collect sensitive personal data. The study adhered to WHO’s ethical guidelines for qualitative studies [14].

## Results

Our analysis of individual interviews with 15 CHC nurses about their interaction and experiences with parents of preschool children in the prevention of childhood obesity yielded 332 codes, 16 subthemes and six main themes. Table 3 presents the subthemes and main themes.

**Table 3:** Classification of main themes and subthemes

**Theme**	**Subtheme**
Assessment of the child’s weight status	Visual inspection only
	Height and weight chart only
	BMI chart
	Manual calculation of BMI
Initiative	Nurses mainly take the initiative
	Concerned parents take the initiative
A sensitive topic	Avoidance or delayed information
	Voluntariness at CHC is important
	No avoidance—a mission
Parental responses	Negative responses
	Positive responses
	Parents deliberately want overweight children
Actions	Health promoting activities
	Preventive activities
Lifestyle patterns	Unhealthy diet habits
	Lack of active play

### Assessment of the child’s weight status

#### Visual inspection only

The nurses visually inspect the children in order to globally evaluate their well-being, physical appearance and mobility. Visual inspection as the only method to assess weight status is unreliable and often inconsistent with objective measurements. Indeed, children who were perceived both by nurses and parents as having normal weight were frequently found to be overweight. Some nurses felt that this reflected an adaption by health care professionals to increasingly more children with overweight or obesity:

It is normal to be slightly overweight, really. We have changed our values somewhat. One doesn’t react quite as quickly as before when children are chubby.

#### Height and weight chart only

CHCs always plot height and weight in a height and weight chart. Some nurses used these charts as the only method for growth assessment. Nurses reported that the weight and height chart had the added advantage of displaying the respective curves. On the other hand, the possible difficulty in establishing the presence of overweight or obesity was a disadvantage. One nurse explained this by saying:

*The weight and height chart is not quite right, because if you measure two above on weight* [i.e. two standard deviations] *and two above on length* [i.e. two standard deviations]*, you would be perfect, but you aren’t, then you are a bit overweight.*

#### BMI chart

The majority of nurses who used the BMI chart thought it was a useful tool in establishing dialogue with parents. Irrespective of parents’ educational background, the chart enabled them to easily understand their child’s weight category. One informant who worked in a suburb with many immigrants, some of who were also illiterate, described the use of the BMI chart:

Well, you know, everyone can’t actually read. We have that problem. But a chart like this, they can understand that if I explain that there is the maximum and this is average and there is the minimum, and so on.

Another advantage of the BMI chart was that no one had to be singled out once it was displayed to all parents, regardless of the child’s weight status. It was also easier to use an objective method that had nothing to do with the nurse’s own opinion:

*Yeah, and then I’m not the one who thought this up. It becomes clearer to the parents* [to be shown the value on the BMI curve]*.*

However, uncertainty about how to use the computerized BMI function was one reason not to use the chart. Many nurses relied on the traditional height and weight chart first and used the BMI chart when they observed a strong indication of either overweight or obesity.

#### Manual calculation of BMI

Nurses who used paper health records had to calculate the BMI manually, a time-consuming procedure that was often done after the health visits. Consequently, parents often did not receive this information until the child’s next health visit.

### The initiative

#### Nurses mainly take the initiative

The majority of nurses said they usually took the initiative:

It was probably me, based on the BMI curve.

Sometimes the parents shared the nurse’s opinion:

Usually, it is one of us who sees it, me or my colleague. That you bring it up for discussion with the parents; what can we do, how did it turn out this way and so on? Then, sometimes the parents join, saying yeah, I might have thought about that, that she has become slightly chubbier.

Health visits for children older than one year of age are scheduled at intervals up to 1–1½ years. This prompted some nurses to take the initiative:

The CHC program. (…) We don’t meet our children. We meet the kids quite regularly during the first year, perhaps at 15 months, then 18 months, then 2½ years, 4 years, and then 5½. So much can happen between those ages.

#### Concerned parents take the initiative

Sometimes parents had a sense that their child had become overweight. Because they were worried, they initiated the dialogue:

I meet many parents who are concerned . . .

In these cases, the nurses were often simply supportive, giving the parents leaflets or connecting them with the dietician.

### A sensitive topic

#### Avoidance or delayed information

Most informants recognized the sensitivity of talking with parents about overweight or, worse, obesity in their child. Some nurses even emphasized that this was an extremely sensitive matter. However, few said they had ever really avoided communicating the topic with parents. One nurse suggested that overweight in children was easier to bring up and discuss if it had not yet accelerated to obesity. On the other hand, when parents were overweight or obese, nurses felt they were implicitly criticizing them for their own overweight and lifestyle. If parents were of normal weight, nurses felt that discussing their lifestyle might accuse them of poor parenting skills.

Nurses sometimes postponed a discussion about unhealthily high BMI. For example, one nurse said,

Well, it has probably happened that, perhaps you have had to stop after you started, because some parents have firmly said ‘no, this is not possible’, and then you have to back out.

Another strategy involved initiating dialogue but not emphasizing the question too heavily to avoid creating a situation where parents would back out. When one nurse had difficulty telling parents directly that their children were in the overweight or obese category, she used the BMI growth chart as a strategy:

*But I feel that when you have the BMI chart, you have so much to benefit there, that you can show it, and we can truly say that now it has increased. No, you can look at it and talk about it, and talk a little about what you could change*.

#### Voluntariness at CHC is important

The trust between the CHC nurse and the parents was an important aspect. The nurses tried to use a defensive approach to avoid pushing parents, which would provoke or jeopardize the relationship. One nurse described this effort as follows:

I show the BMI charts and all the other charts, the whole lot, but still, they are here voluntarily, and if I’m not listened to, then I have to wait.

#### No avoidance—a mission

Several nurses said that they felt it was their duty to talk about a child’s obesity, and two nurses even stressed that it was their mission to be advocates and to keep the interests of the child in focus:

I do not think that I have avoided it, because my task it is to ensure that the children feel as well as possible, and get a good start in life, and then I have 6 years together with them in the CHC, and that is not much. You know, a lot happens between the ages of 2 1/2 years and 6 years, and if I see something, then it’s my responsibility.

### Parental responses

#### Negative responses

Nurses described parents who were not receptive to information about their child’s weight status, possibly due to denial or feeling offended. When nurses offered such parents extra visits, some did not come. Occasionally, some parents became angry or turned to another CHC:

One mother stated very clearly that “I find it so hard to come to you because you always bring this up.” She got up and left; the father remained. I sat silent for a while, then I said: “I feel really sad that it has become like this, because my mission here is to help the children.” And now it turned out that she registered at another CHC, and hopefully they got another CHC nurse who could get into a good relationship with the mother.

#### Positive responses

The nurses encountered parents who changed their attitudes and became more receptive. Some parents responded by becoming a little sad, but they still attended the next health visit. After parents had reconsidered their conversation with the nurse, they became more responsive, gradually changed their mind, and started thinking about the family’s habits. One father who responded this way later told the nurse that he had obesity as a child and had now decided to prevent his son from having the same experience. He spent more time outdoors with his child and created conditions where the child could be more active.

The nurses also described parents who sought help. Another group of parents who had not initiated the discussion immediately accepted the offer of extra visits or accepted a nurse’s proposal to refer them to a pediatrician or dietitian for counseling. Nurses described these situations as successful. On these occasions, nurses usually only needed to support the parents, because the parents often already knew what type of family habits needed to be changed:

They embrace what you talk about, changing the diet and trying to assimilate the tips and advice that I have given. They know best themselves what the problem is, so it is really about me confirming them, and trying to help them with a solution that they actually might know themselves. The easiest ones are the parents who say ‘help me’. They’re definitely the easiest.

#### Parents deliberately want overweight children

Some parents did their best to make their children overweight, often due to an influence of older relatives or an irrational and unfounded concern:

But we may think, and this is a constant nagging, you have these quite normal, or just slightly too chubby babies, which the parents are not happy with. They want them to be even chubbier. But, sometimes there are actually parents who want their 1- 3-year-olds to be chubby. You know, quite often I have seen that slim 2½ -, 3-year-olds, will be obese 4-or 5-year-olds. And then the parents are satisfied, because then the kid eats. And then, it is hard to reach them with the information that now it has gone too far.

### Actions

#### Health promoting activities

Activities include repeated measurements of weight and height for all children at every health visit and a demonstration of growth for all parents. Because nurses take a holistic approach to the health visit, they use growth monitoring as a platform for health promoting activities at large. One informant commented this way:

We follow the family with dietary advice all along, so really, you shouldn’t find a 4-year old child with obesity without having done anything before.

Another routine in standard CHC procedures is to a questionnaire on food and activity habits given when the child is 18 months and 2½ years of age. This form gives an opportunity for structured conversation regarding parental concern about their child’s weight, physical activity, sleep and food habits. The majority of informants said that they held parent group meetings at their CHC, but only before the child was one year of age. At two CHCs, both located in areas with a large immigrant population, nurses had difficulty gathering the parents for parent groups.

#### Preventive activities

CHCs offered extra visits to children with overweight or obesity, where CHC nurses monitor growth and BMI development and discuss family lifestyle and routines. All nurses mentioned this kind of preventive work. As part of the treatment program, the nurses also offered a referral to the pediatrician for children with obesity.

*If the child has obesity*, *then I suggest a doctor’s appointment.*

A majority of nurses also mentioned that they could refer the family to the dietician.

Nurses stressed the importance of feeling confident that the pediatrician would support their decisions:

And what we’re working hard on, everyone who works at the CHC, and those who work in the children’s team, is that we try to talk the same language, that we do not say different things, because it gives a sense of insecurity.

However, two nurses had encountered physicians who dismissed childhood obesity as harmless and reassured parents that the child would outgrow it. These nurses felt their efforts in motivating the parents were in vain.

### Lifestyle patterns

#### Unhealthy diet habits

Nurses mentioned parents’ lack of cooking skills. One informant showed parents oatmeal packages so they would be able to recognize them in the stores. She wanted parents to cook their own porridge for the children:

I who think that you should cook your own oatmeal for your children at home, I have given up. Everyone buys powder porridge, so you have to accept that they do that.

Some parents without cooking problems had the habit of serving portions that were too large, increasing the risk of accelerated weight gain, especially if the child had a good appetite. Here, the challenge for the nurse was the dual task of (i) supporting parents’ good skills and habits and (ii) suggesting that parents reduce portion size and not serve the food from the table:

*They had no bad food, really, but too much food. They cook a lot of food and place it on the table, and if it is a child with a good appetite, then no wonder it eats too much. Many big kids eat large portions*.

Another aspect involved parents who gave sweets to their children out of pity:

Perhaps you may talk about sweets and candy, and some introduce that really early, and you might ask why, ‘well, it’s such a pity for him if we eat candy and he can’t get any’, but he does not know anything else, and then you feel that they think in the wrong way as parents.

A special category of parents included those who had experienced a difficult neonatal period and unknowingly continued to feed their children too often or too much due to lingering concern:

Then it could be these children who were very small when they were newborn, and perhaps breastfeeding did not work and later on, they were slow to start eating small portions of real food.

Another potential reason for increased weight gain involved parents who served gruel in baby bottles to older children. Gruel is easy to prepare and an older child can hold the bottle itself, making it easy to serve at night or before the child goes to day care. One nurse said that she always asked parents of children with overweight or obesity if they gave the child gruel at night, because, given too often, gruel easily resulted in an overload of energy.

#### Lack of active play

Nurses described parental unawareness of promoting daily physical activity and avoiding sedentary behavior. One informant said that it was not unusual for three to four-year-old children to sit in a stroller rather than walk. Some parents unconsciously hindered their children from active play because they were concerned that their children would become dirty. Others thought that the children had already been active enough at kindergarten so it was not necessary for them to play at home. Encouraging outdoor and active play rather than sedentary play was a constant discussion theme, one that was stressed by a majority of the nurses.

### Grouping subthemes into facilitators and barriers

We divided the subthemes into facilitators and barriers. Facilitators included the following subthemes: the BMI chart, concerned parents take the initiative, nurses mainly take the initiative, no avoidance—a mission, positive parental responses, health promoting activities and preventive activities. Barriers included visual inspection only, height and weight charts only, manual calculation of BMI, avoidance or delayed information, the importance of voluntariness at the CHC, negative parental responses, parents who deliberately want their children to become overweight, unhealthy diets and lack of active play. In Table 4 we summarize the subthemes of barriers and facilitators according to the owner of the issue of obesity versus the owner of the solution, i.e. the nurse or the parent. The table is inspired by Söderlund et al. [25].

**Table 4:** Facilitators and barriers in nurse/parent interaction at child health centers

	**Obesity prevention issue/solution owner**	
	**Nurses**	**Parents**
	BMI chart	Concerned parents take the initiative
	Nurses mainly take the initiative	Positive responses
**Facilitators**	No avoidance—a mission	
	Health promoting activities	
	Preventive activities	
	Visual inspection only	Negative responses
	Height and weight chart only	Parents deliberately want overweight children
**Barriers**	Manual calculation of BMI	Unhealthy diet habits
	Avoidance or delayed information	Lack of active play
	Voluntariness at CHC is important	

## Discussion

The findings reported here represent CHC nurses’ view of their interactions with parents of preschool children with overweight or obesity. Interviews were held in widespread geographical areas of CHCs comprising a variety of Swedish demographic and socioeconomic characteristics. By default, CHCs in Sweden have a long tradition of growth monitoring and a very high uptake rate (i.e. > 99% of children visit the nurse’s office at least six times) [9], which gives obesity prevention a key position at CHCs. Consequently, assessment of a child’s weight status was a central theme in the topic guide. Our results showed that all CHC nurses used the traditional weight-for-height chart, which plots two different growth curves. However, nurses did not always use the recommended BMI chart [10,11]. Those who used the BMI chart reported several benefits. Weight problems were easier to detect compared to height and weight charting, which concurs with the results of other studies [26]. In addition, the BMI chart is easy to understand by parents of all educational backgrounds. Visualization of overweight or obesity with the BMI chart favors objective communication. Consistent with previous qualitative studies, nurses in our study were able to set aside their personal and subjective perceptions about a child’s weight status [18]. Nurses applied a participatory approach, displaying children’s charts and informing parents about their child’s growth pattern. In a 2005 survey of CHC nurses (n = 270, response rate 80%) in our study region, only 14% reported using the BMI chart. Consequently, the county of Västra Götaland decided to recommend use of the BMI chart [27]. However, the CHCs in this study did not seem to follow those recommendations fully, possibly due to a lack of quality assurance methods or a lack of computers or computer skills.

Health professionals (e.g. CHC nurses) are ideally placed to identify children at risk for obesity and to play an active role in obesity prevention [28]. By plotting a child’s BMI at all health visits and using any change in BMI to identify excess weight gain, CHC nurses can activate obesity prevention strategies [28,29]. Despite strong recommendations by the American Academy of Pediatricians, the BMI chart remains underused [26,30].

Several studies also underline the importance of early obesity prevention [2,5,6,31,32]. Whitaker et al. [31] showed that children with obesity under the age of three years are at low risk of obesity in adulthood. Harrington et al. [32] concluded that the critical period for preventing childhood obesity is during the first two years of life. In our study, nurses described their CHC standard procedures to administer a questionnaire regarding the child’s development, food and activity habits at 18 months and 2½ years of age as a valuable opportunity and a facilitator for confidential and structured dialogues with all parents of children under the age of three years. Thus, the risk that parents would feel offended or react negatively decreases since, at that age, the focus is not on the child’s BMI but on lifestyle habits and behaviors.

Another theme involved determining who initiated discussions about an issue regarding a child’s weight status. Nurses responded that they usually took the initiative, although some concerned parents also initiated the discussion. Because parents have a particular interest in their child’s growth, it is reasonable to expect that they would initiate a discussion if the child gains weight too rapidly. However, it may be unrealistic to assume that parents who lack an accurate perception of their child’s overweight or obesity could initiate a discussion. Several studies reported that parents of children with overweight or obesity underestimate their children’s weight status [15,16]. The Identification and Prevention of Dietary- and Lifestyle-Induced Health Effects in Children and Infants study (IDEFICS) investigated two to nine-year old children and their parents in eight European countries. Between 50% and 77% of parents of children with overweight (n = 1,968) perceived their children to be of normal weight and 70% of the parents of children with obesity (n = 1,072) perceived their children as only “slightly too overweight” [16]. However, it is unknown whether these parents had received accurate information about their child’s weight status at a CHC or the school health service. Because CHC nurses in Sweden have access to the child’s growth charts, they can objectively judge the child’s weight status and communicate their findings to the parents, who lack access to the charts. Another advantage at the preschool age is that parents are always present when a nurse measures their child’s growth at CHC, which facilitates the communication of the findings, as compared to school aged children when parents are not present when measurements are made and need to receive notification by other means.

Several studies have observed that childhood obesity is a sensitive topic because healthcare professionals fear offending parents [17,18,33]. Our study supports these findings. Nurses sometimes avoid the word obesity, using “overweight” to describe both overweight and obesity, possibly due to a desire to be empathetic. This approach may confuse the definition of overweight and obesity and make communication with parents less clear. Nurses also reported that they sometimes retreated because of parents’ reactions. Others emphasized that avoidance was never an option, even when parental responses were strained. They explained that they relied on ethical values and stressed children’s right to a healthy start in life. One nurse referred to the United Nations Convention on the Rights of the Child (UNCRC) which stipulates “the right of the child to the enjoyment of the highest attainable standard of health” (art. 24) [34]. These values strengthened the nurses’ resolve to initiate a discussion with parents because they saw this as their mission.

Parental responses and nurses’ attitudes indicated that obesity prevention at CHC is both an organizational and an emotional issue. Our results discerned two subthemes of parental responses: (i) cooperative and help-seeking parents who easily benefited from CHC resources (i.e. positive responses) and (ii) parents who responded negatively, did not attend extracurricular health visits or went to another CHC. The lack of options for reaching these parents revealed an opportunity gap in supporting their children. Good health and equal care on equal terms for the entire population is expressed in Swedish law [35]. Moreover, Sweden has ratified the UNCRC [34]. Parental cooperation with lifestyle changes is fundamental to the success of obesity prevention [29]. CHCs must reach out to parents who respond negatively when nurses inform them about their child’s obesity. According to Mikhailovich and Morison [19], healthcare professionals should expect a wide range of parental responses and a critical period in communicating “difficult” news. Parents may fear that their child will be stigmatized. Therefore, healthcare providers need to create an environment of support and partnership [19].

Some parents deliberately struggle to make their toddler overweight. Nurses explained that such parents believe that overweight represents health and successful parenting, in line with Baughcum et al. [36]. In agreement with others [16,37], some nurses in this study also interpreted parents’ desire for a “chubby child” as an irrational concern that their child might become underweight.

CHC actions were integrated into the regional CHC program [10]. The nurses aimed to give nutrition advice at all health visits and to follow the development of the child and the well-being of the family. Therefore, growth measurements are not only a technical procedure but also part of a holistic view of the child and family in the context of the family’s social circumstances. A recent study of CHCs in western Sweden reported that nurses expressed uncertainty about determining a child’s weight status compared to BMI and also about counseling parents when their child was overweight or obese [38]. In yet another Swedish CHC study [39], audiotapes of conversations with parents revealed infrequent attention to dietary and physical activity behaviors. The study ranked 23 topics of conversation according to the median proportion of total session time. In descending order, dietary habits ranked at number 4 (9.5%) and physical activity behaviors first at number 14 (3.5%).

The nurses followed basic CHC guidelines and referred children with obesity to a physician and frequently also to a dietitian [10]. Parent groups at CHC were only held for parents of children younger than one year. When children began to eat the same food as the family, CHCs could no longer gather parents easily and inform them about healthy eating habits, often because parents had returned to work and had less time. Moreover, CHCs had difficulty implementing parent groups in areas with high immigration rates and low socioeconomic status because parents often did not attend the sessions. This was disclosed through the purposive sampling selection of a diverse geographical and demographic background. This is also a barrier because health-promoting activities must equally reach all types of families. In addition, the prevalence of obesity in Sweden is higher in areas with high immigration [40]. In different areas of Stockholm, families with low purchasing power strongly correlate with obesity in four-year-old children born in 2006 [41]. Wallby and Hjern [42] showed that low income and foreign born mothers had lower rates of participation in parental groups at CHC in a Swedish county. In the Swedish IDEFICS health survey, families characterized by single parenthood, foreign background, and low education and income were underrepresented compared to the general population [43]. Therefore, CHCs need to strengthen health promoting and prevention actions for young children to ensure that such families receive access that equals that of affluent families and cooperative parents.

In the theme of lifestyle patterns, our results showed that parents are influenced by the obesogenic environment, which corresponds to the WHO population strategy [1] that seeks to shift the responsibility from the individual to societal stakeholders in tackling health risks. Even though the majority of parents try to do their best, they may need to be “super parents” to resist the influences of today’s obesogenic environment [44,45]. In the present study, several nurses observed that food marketing heavily influences parents’ choices of industrialized and convenience foods. This finding concurs with parental views about factors that influence eating behaviors in their two to eight-year old children, as expressed in focus group discussions held in IDEFICS [46]. Our nurses observed that many parents underestimate children’s need for active play and allow too sedentary activities. For example, it was not unusual to see children sitting in a stroller when they were old enough to walk, which concurs with IDEFICS’ parental focus group discussions about children’s physical activity [47], i.e. parents frequently mentioned that their children did not like to walk. However, it is important to recognize the multicomponent and complex web of causes of the obesity epidemic [48]. There are several opportunities for obesity prevention, not only at the individual level but also at the national and community levels: for example, new and improved food labeling, reduced advertisements for unhealthy foods and beverages, increased access to green spaces and physical activity and social marketing on what and how much to eat and how to reverse the culture of long work days [48,49].

### Strengths and limitations

A strength of this study is that 15 nurses from different geographical parts of Sweden, which comprise a variety of population demographic characteristics, gave their view of health promotion and obesity prevention interactions with parents. The majority also had long-standing experience as CHC nurses and can be considered experts in the field. Our nurses offered full and vivid descriptions of the predetermined themes. The professional background of the interviewer (SR), a pediatric nurse with practical experience of working with parents and children as well as professional concepts and practices, may have strengthened confidence during the interviews and contributed to the sharing of informants’ experiences. We assured rigorous analysis (Table 2) by preparing and organizing the content analysis process, following a structure that progressed from meaning unit to code, subtheme and theme. The extended and supportive findings of others increased the strength of our directed and deductive approach [21,22]. Conformability was ensured because the authors agreed with the findings and brought their unique perspectives and experience to the study. The research group critically discussed all categorizations into subthemes and themes.

To some extent, the limitations of this study are shared by other qualitative studies as part of methodological constraints. For example, those who decided to participate were presumably motivated to give their full view of the subject of interest, while those with a contrary view were not heard. Furthermore, because parents did not participate in the study, their voices were unheard. Consequently, parents could not dispute or agree with statements about the parents. Consequently, the parental views reported in this study are told through the eyes and perspective of the nurses. However, we still believe that it is possible to transfer their perspectives to similar settings and conditions.

## Conclusion

The systematic organization of CHCs in Sweden, which by default conduct growth measurements at all health visits, provides a favorable opportunity to prevent childhood obesity. However, inconsistent use and lack of quality assurance regarding the recommended BMI chart may hinder identification of children with overweight or obesity. Nurses who used the BMI chart considered it a facilitator for greater recognition of a child’s weight status, that it was easy to explain and display to parents and was an objective measuring tool. Nurses viewed obesity as a sensitive topic, and nurses were the main initiators of discussions about a child’s overweight or obesity. The BMI chart allows improved identification of overweight and obesity in children, but remains underutilized at CHCs in southwest Sweden.

## Abbreviations

BMI: Body mass index; CHC: Child health center; IDEFICS: Identification and prevention of dietary- and lifestyle-induced health effects in children and infants study; UNRCR: United Nations convention of the rights of the child; WHO: World Health Organization.

## Competing interests

The authors declare that they have no competing interests.

## Authors’ contributions

All three authors contributed to the analysis and drafting of the manuscript. SR designed the study, conducted the interviews, and made the transcripts and initial analysis. All authors critically discussed each step of the analysis. The final manuscript was read and approved by all authors.

## Pre-publication history

The pre-publication history for this paper can be accessed here:

http://www.biomedcentral.com/1472-6955/12/27/prepub
